# Impact of cardiac surgical timing on the neurodevelopmental outcomes of newborns with Complex congenital heart disease (CHD)

**DOI:** 10.3389/fped.2023.1003585

**Published:** 2023-03-23

**Authors:** Marien Lenoir, Thibault Beretti, Benoit Testud, Noémie Resseguier, Kim Gauthier, Virginie Fouilloux, Célia Gran, Florent Paoli, Fedoua El-Louali, Philippe Aldebert, Julie Blanc, Camille Soulatges, Sarab Al-dybiat, Guillaume Carles, Chloe Wanert, William Rozalen, Stéphane Lebel, Sophie Arnaud, Dominique Santelli, Chloé Allary, Marianne Peyre, Isabelle Grandvuillemin, Clotilde Desroberts, Myriem Belghiti Alaoui, Farid Boubred, Fabrice Michel, Caroline Ovaert, Mathieu Milh, Clément François, Béatrice Desnous

**Affiliations:** ^1^ Division of Paediatric Cardiac Surgery, APHM La Timone, Marseille, France; ^2^ Département de Pédiatrie, Division de Neurologie, Hôpital de La Timone, Marseille, France; ^3^ Department of Neuroradiology, APHM La Timone, Marseille, France; ^4^ CEMEREM, APHM La Timone, Marseille, France; ^5^ Aix-Marseille University, Support Unit for Clinical Research and Economic Evaluation, AP - HM, Marseille, France; ^6^ Department of Paediatric Neurology, APHM La Timone, Marseille, France; ^7^ Department of Paediatric Cardiology, APHM La Timone, Marseille, France; ^8^ Department of Paediatric Anesthesia and Intensive Care Unit, APHM La Timone, Marseille, France; ^9^ Department of Neonatology, APHM La Conception, Marseille, France; ^10^ Aix Marseille Univ, CNRS, LPL, Aix-en-Provence, France; ^11^ INSERM U1106 Institut de Neurosciences des Systèmes, Marseille, France

**Keywords:** bayley IV, congenital heart disease, white matter injuries, cardiac surgical timing, neonates, neurodevetlopmental outcomes

## Abstract

**Background:**

More than half of infants with complex congenital heart disease (CHD) will have a neurodevelopmental disorder of multifactorial causes. The preoperative period represents a time-window during which neonates with complex CHD are in a state of hypoxia and hemodynamic instability, which fosters the emergence of brain injuries and, thus, affects early brain networks and neurodevelopmental outcomes. Currently, there is no consensus regarding the optimal age for cardiac surgery in terms of neurodevelopmental outcomes, and its definition is a real challenge. Our aim is to determine the relationship between cardiac surgical timing and long-term neurodevelopmental outcomes for various types of complex CHD.

**Methods:**

We hypothesize that earlier surgical timing could represent a neuroprotective strategy that reduces perioperative white matter injuries (WMIs) and postoperative morbidity, leading to improved neurodevelopmental outcomes in infants with complex CHD. Firstly, our prospective study will allow us to determine the correlation between age at the time of surgery (days of life) and neurodevelopmental outcomes at 24 months. We will then analyze the correlation between age at surgery and (i) the incidence of WMIs (through pre- and postoperative MRIs), (ii) postoperative morbidity, and (iii) the duration of the hospital stay.

**Implications and Dissemination:**

This research protocol was registered in the Clinical Trial Registry (National Clinical Trial: NCT04733378). This project aims to help launch the first Neurocardiac Investigation Clinic in Marseille — AP-HM — to propose an overall personalized monitoring and treatment program for patients operated on for complex CHD.

## 1. Introduction

Congenital heart disease (CHD) is the most common birth defect, affecting 9 neonates out of every 1,000 live births (i.e., nearly 1% of births) (1). Half of these children have complex CHD requiring cardiac surgery during the first months of life (1). Additionally, more than 50% of them have a neurodevelopmental disorder resulting from multiple additive, collinear, and interacting risk factors, including genetic variants, parental education, fetal circulatory disorders, intraoperative factors, and postoperative complications (2). The current prognosis for neonates with complex CHD warrants the development of prevention strategies aimed at improving their neurodevelopment outcomes and, thus, their quality of life. Cardiac surgery with its intrinsic risk factors, such as the duration of extracorporeal circulation (EC) or the realization of circulatory arrest, does not determine the neurodevelopmental outcomes of these patients. Indeed, the preoperative period represents a window of time during which neonates with complex CHD are in a state of hypoxia and hemodynamic instability, fostering the emergence of brain injuries (3–5) and, thus, affecting early brain networks (6) and neurodevelopmental outcomes.

For patients with TGA (Transposition of Great Arteries) or HLHS (Hypoplastic Left Heart Syndrome), later cardiac surgery leads to a higher incidence of white matter injuries (WMI) (7, 8), which is a well-known prognostic factor for motor (9–12), language (11), and cognitive (10, 12, 13) impairment, as well as behavioral issues (14). Additionally, Anderson et al. (15) demonstrated that conducting the arterial switch operation (ASO) on neonates with TGA before the third day of life reduced the major postoperative morbidity of these patients in terms of neurological (occurrence of seizures, ischemic stroke), cardiological, and infection complications. Patients with left ventricle hypoplasia operated on later in the neonatal period also presented higher postoperative morbidity (16). Similarly, patients with TGA given the ASO after 2 weeks of life experienced impaired perioperative brain growth and slowed language development (17). Conversely, a major hypotrophy (weight < third percentile) or hemodynamic instability can delay the surgery, which may then be considered too risky. The fetal, pre and intraoperative risk factors of brain injuries are listed in the Table 1. All postoperative morbidities (Table 2) are deemed as potential risk factors for brain injuries.

**Table 1:** Foetal and preoperative risk factors of brain injuries.

	Foetal period	Preoperative period	Intraoperative period
Risk Factors of Brain injuries	Gestational Age	Postnatal diagnosis	CPB duration (min)
	IUGR	Lower arterial oxygen saturation	Aortic clamp time (min)
	Brain volume	Lower apgar scores at 5 min	DHCA (min)
			Selective cerebral perfusion (min)
	Brain immaturity	Abnormal aEEG background pattern	Low Flow
		Longer time to surgery	
		Brain lactates	
		Low flow	
		Male sex	

CPB, Cardiopulmonary bypass; DHCA, Deep hypothermia with circulatory arrest; IUGR, Intrauterine growth restriction; aEEG, amplitude EEG.

**Table 2:** Classification of post-op morbidities.

Morbidity category	Morbidities	
Cardiac / Respiratory	• Cardiac arrest, arrhythmias, JET • Low cardiac output syndrom • Extracorporeal membrane oxygenation • Post-op high-output failure • Re-intubation for acute respiratory distress	• Neonatal necrotizing enterocolitis • Systemic vein thrombosis • Acute renal deficiency with dialysis • Delayed sternal closure • Length of intensive care unit stay
Infectious	• Septicemia, fungemia • Infection of the surgical site, mediastinitis • Endocarditis	• Meningitis • Pneumonia
Neurological	• Ischemic cerebrovascular accident • Hemorrhagic cerebrovascular accident	• Epileptic seizure • Cerebral thrombophlebitis
Surgical	• Recurring diaphragmatic paralysis/paresis • Definitive AV block with device • Acute hemorrhaging requiring revision surgery	• Cardiac tamponade • Unplanned revision surgery • Chylothorax

There is no consensus regarding the optimal age for surgery in terms of morbidity and neurodevelopmental outcomes, and its definition is a real challenge. Importantly, at the present time, there are no established rules for determining the timing of surgery in our department. For the most common CHD types, the TGA are corrected during the first ten days of life. There is a more pronounced degree of variation for HLHS. This common practice won't be changed for our observational study.

Our aim is to determine the relationship between surgical timing and long-term neurodevelopmental outcomes for various types of complex CHD. Similarly to Anderson et al. (15) with postoperative morbidity, we aim to determine a cut-off point for surgical timing that will improve neurodevelopmental outcomes. For each type of CHD, we aim to determine a cut-off for surgical timing based on postoperative morbidity and neurodevelopmental outcomes. We hope that our findings will contribute to the development of surgical timing guidelines in the future.

### 1.1. Hypotheses

We propose an open-design, observational longitudinal study in which we hypothesize that earlier cardiac surgery may constitute a preoperative neuroprotective strategy and improve the neurodevelopmental outcomes of neonates with complex CHD.

### 1.2. Main objective

The main objective of this study is to assess the correlation between age at surgery (days of life) of neonates with complex CHD and neurodevelopmental outcomes at 24 months, assessed by the scores obtained on the subscales of the Bayley Scale of Infant and Toddler Development®, Fourth Edition® (Bayley-IV test) exploring motor skills, cognition, and language (18).

Our aim is to determine the optimal threshold for the age at surgery that discriminates delayed neurodevelopment / normal neurodevelopment according to the main criterion, i.e. neurodevelopment at 24 months according to the Bayley-IV scale (score <80 vs. ≥80).

### 1.3. Secondary objectives

The secondary objectives of our study are to evaluate the correlation between age at surgery (days of life) of neonates with complex CHD and the following outcomes:

i. the presence and severity of WMIs observed on preoperative and postoperative brain MRI;
ii. postoperative morbidity;
iii. the duration of the postoperative hospital stay;
iv. neurodevelopmental outcomes, including clinical pediatric neurological assessment (4 months, 12 months, and 24 months) and functional motor skill assessment using the Alberta Infant Motor Scale (AIMS; 4 months and 12 months).

We will also assess the moderating role of demographic characteristics and pre-, intra-, and postoperative factors on the following outcomes:

i. the frequency of pre-, and postoperative WMIs;
ii. postoperative morbidity;
iii. neurodevelopmental outcomes, including clinical pediatric neurological assessment (4 months, 12 months, and 24 months), functional motor assessment using the AIMS (4 months and 12 months), and the Bayley-IV test (24 months).

## 2. Methods

### 2.1. Recruitment

Recruitment will be conducted on a prospective cohort (*n *= 100) of neonates with complex CHD requiring cardiac surgery with cardiopulmonary bypass CPB within the first 2 months of life and receiving a pre-operate and postoperative brain MRI, standardized neurological assessments at 4 months, 12 months, and 24 months, and a neuropsychological assessment with a Bayley-IV test at 24 months.

### 2.2. Inclusion criteria

In this study, we will recruit infants aged 2 months or less with complex CHD requiring surgery with extracorporeal circulation during their first 2 months of life at the La Timone Enfants Hospital in Marseille. The included patients should be born full-term (at more than 37 weeks). The eligible complex CHDs correspond to the anatomical classification of Clancy et al. (19), (Table 3).

**Table 3:** Classification of complex congenital heart diseases selected for the study, according to Clancy *et al*. (19).

Classification Number	Anatomic Classification (10)	Complex CHD
I	Two-ventricle heart without arch obstruction	TGA with intact ventricular septum
		TGA/VSD
		Tetralogy of Fallot/PA
		Truncus Arteriosus
		TAPVR
II	Two-ventricle heart with arch obstruction	TGA/VSD/CoAo
		VSD/CoAo
		VSD/Interrupted aortic arch
III	Single-ventricle heart without arch obstruction	Unbalanced AVCD
		Tricuspid atresia
		Mitral atresia
		Heterotaxy
		Double-outlet left ventricle
		Double-outlet right ventricle
		Single ventricle (other)
IV	Single-ventricle heart with arch obstruction	HLHS

PA, Pulmonary Atresia; AVCD, Atrioventricular Canal Defect; VSD, Ventricular Septal Defect; CoAo, Coarctation of the Aorta; HLHS, Hypoplasia of Left Heart Syndrome; TAPVR, Total Anomalous Pulmonary Venous Return; TGA, Transposition of the Great Arteries.

For all patients, we must obtain informed consent from both parents (except in special cases justifying consent from just one parent) or from legal representative(s). The parents or legal representatives must be able to read and speak French. Additionally, the included patients will need to be affiliated with French social security.

### 2.3. Exclusion criteria

Patients will be excluded from this study in cases where they (i) have not received any preoperative or postoperative brain MRI due to their poor state of health, because the parents refused, or for another reason; (ii) have their consent withdrawn.

### 2.4. Non-inclusion criteria

Patients will not be included if (i) birth weight is less than 2 kilos and/or gestational age less than 37 weeks (ii) if they have a CCHD not requiring cardiac surgery with CBP during the first 2 months of life And (iii) if they have a proven chromosomal abnormality or genetic syndrome associated with their CCHD.

### 2.5. Procedure overview

**Figure 1:**
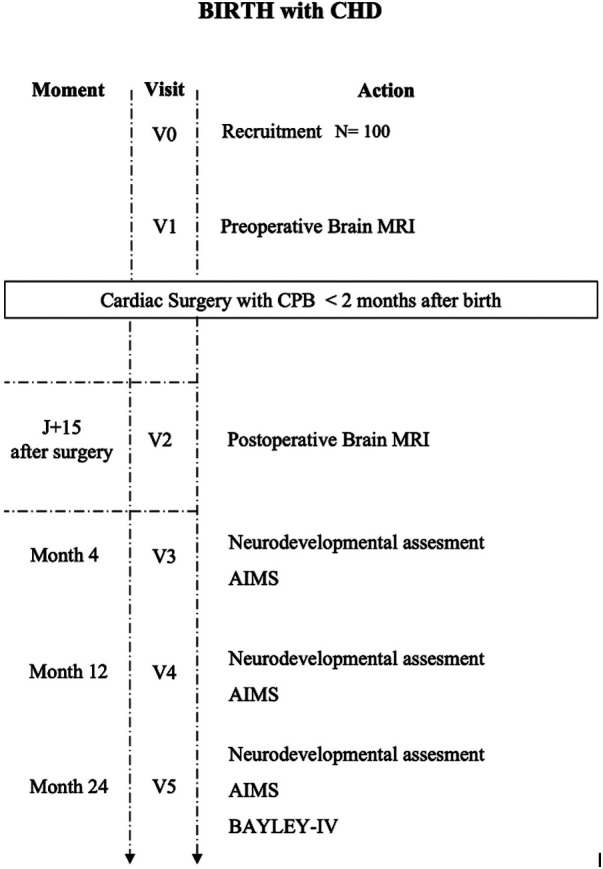
24-month monitoring of patients born with CCHD operated on within the first 2 months of life. CPB, Cardiopulmonary bypass; CHD, Congenital Heart Disease; MRI, Magnetic Resonance Imaging; AIMS, Alberta Infant Motor scale; BAYLEY scale fourth edition.

Each recruited patient will participate in the study for 24 months (Figure 1). Following the recruitment (visit 0, V0) conducted in the neonatal period before cardiac surgery, a preoperative brain MRI will be performed before surgery (visit 1, V1) if the health of the neonate allows for this. During the 15 days following the surgery, a postoperative brain MRI is performed (visit 2, V2). Visits 1 and 2 aim to assess preoperative brain injuries and those that appear or worsen postoperatively. The preoperative MRI should not delay cardiac surgery. Visits 3 (V3), 4 (V4), and 5 (V5) will involve neurodevelopmental assessments at 4 months, 12 months, and 24 months, respectively, conducted by a single child neurologist (Dr. Desnous) and the Alberta Infant Motor Scale (AIMS) assessment at 4 months and 12 months. These assessments will be scheduled and conducted during conventional follow-up cardio-pediatric appointments. During visit 5 (V5), at 24 months, a standardized neuropsychological assessment will be performed using the Bayley-IV test.

#### 2.5.1. Prenatal period

The La Timone Enfants University Hospital (Marseille) is the regional reference center for pediatric cardiac surgery in the Provence Alpes Côtes d'Azur (PACA) region, south-east of France. As part of pregnancy monitoring, and in the event of abnormal cardiac measurements, parents are referred to an ultrasound physician specializing in fetal cardiac ultrasounds. When the parents decide to continue the pregnancy, they meet the cardiac surgeon, who explains the details of the operation to be performed after birth and present the study to the parents to explain the objectives, constraints, and procedures.

#### 2.5.2. Post-partum period

After birth, which takes place in a level 3 maternity ward, patients are admitted to the Pediatric Intensive Care Unit (PICU) at La Timone Enfants Hospital for clinical stabilization, cardiac assessment, and emergency procedures. If the parents or legal representatives were informed of this study in advance and agreed to participate, they would be asked to sign the consent form with the child neurologist. In the unlikely event that the parents could not be informed prior to the beginning of the study, and where the study's procedures would be compatible with treatment within the framework of the patient's care, consent will be obtained upon the child's admission to the PICU but after a 24-hour reflection period.

### 2.6. Brain MRI

A pre- and postoperative brain MRI will be performed on a clinical 3 T Vida MRI system (Siemens Medical Systems) using a 64-channel head coil. Protocol will include 2D T1-weighted, T2-weighted, and diffusion-weighted imaging and 3D isotropic T1-weighted imaging to highlight preoperative brain injuries, as well as those that appear or worsen postoperatively. All MRI sequences use thin cuts (1 mm), and the entire MRI protocol is adapted for neonates and infants to optimize contrast. The preoperative brain MRI will be performed just prior to surgery and the postoperative one within 15 days following cardiac surgery.

The neonate will be comfortably secured in an adapted cocoon after administration of a normal food ration, without general anesthesia or injection of a contrast product. The completion time for the MRI is between 30 and 45 min.

### 2.7. Genetic workup

A biobank called SEA-CARREG has been established by the cardiology department, and all recruited patients will have a genome.

### 2.8. Neurodevelopmental assessment

Neurodevelopmental evaluations will take place between 4 and 24 months to evaluate cognitive and language outcomes. This neurodevelopmental assessment will be performed by a single child neurologist (BD) at 4, 12, and 24 months and will be based on the AIMS and the Bayley-IV.

#### 2.8.1. AIMS - Alberta infant motor scale

The AIMS assessment will be performed at 4 months and 12 months. The AIMS is a standardized tool allowing the neuromotor assessment of infants from 0 to 18 months old and the identification of children with abnormal motor development. The scale consists of 58 items exploring the functional motor skills of infants in sitting, standing, prone, and supine positions. The interpretation of the results for each position and the overall score will be completed using percentile ranks according to the age of the infant. These assessments will be scheduled and performed during conventional cardio-pediatric follow-up visits.

#### 2.8.2. Bayley-IV

A standardized neuropsychological assessment using a Bayley-IV test will be performed at 24 months. The Bayley-IV test is used to assess children aged 1 to 42 months. This is a standardized scale (mean = 100, standard deviation = 15), which has demonstrated good sensitivity in detecting neurodevelopmental disorders in patients with CCHD. The items are organized according to the level of difficulty. The scale contains five subscales, three of which (cognition, motor skills, language) require interaction with the child and will be used for the analysis. The two other subscales will beassessed through questionnaires completed by the parents or legal representatives and relate to the socio-emotional sphere, communication of needs, and self-regulation for the first part and adaptive behavior, communication, and autonomy for the second part. These results will not be retained for analysis. This assessment will be scheduled and performed during a conventional cardio-pediatric follow-up visit.

### 2.9. Morbidities

An analysis will be conducted for postoperative morbidities (see Table 2), including cardiac, hemodynamic, surgical, infectious, and neurological morbidities. Each of these complications will be analyzed independently.

Perioperative demographic and clinical data, including postoperative complications, will be collected retrospectively from prospectively completed medical files. We are particularly interested in the following data:

(i) demographic, including gender, education level, and economic status of the parents;
(ii) preoperative, including whether there was a prenatal diagnosis of complex CHD, gestational age at birth, birth weight, head circumference at birth, Apgar score at 5 min, type of complex CHD, whether a Rashkind procedure was performed, the type of ventilation and its duration in days, hypotension requiring treatment, cardiac arrest, and arrhythmia.
(iii) intraoperative, including age and weight at the time of surgery, hematocrit in the operating room, type of surgery, CPB time in minutes, selective cerebral perfusion time, and circulatory arrest time in deep hypothermia in minutes.
(iv) postoperative, including ventilation (non-invasive, mechanical), duration in days, anesthesia, sedatives and exposure to antiepileptics, medication names and number of days used, and total and postoperative length of their stay in days.

## 3. Statistical analysis

### 3.1. Analytical strategy

The data analysis will be performed blindly using R -3.4.1 for Windows.

#### 3.1.1. Sample size

Two previously published studies that reported a significant correlation between age at surgery and the incidence of WMIs included 26 and 37 patients, respectively (7, 8). Based on the active file of patients with complex CHD operated on each year in Cardiac Surgery at the La Timone Enfants Hospital, the total number of subjects included will be 100. With this number of 100 included patients and according to the values of (i) a 5% bilateral alpha risk and (ii) 80% statistical power, we estimate the minimum correlation coefficient between age at surgery and Bayley-IV test scores as 0.39.

According to the same hypotheses (5% bilateral alpha risk and 80% statistical power), and based on the intended number of patients recruited for each (Clancy) CHD subtypes, the minimum correlation coefficient that could be highlighted between age at surgery and Bayley-IV test scores is:

*r* = 0.38 for class I, two-ventricle heart without arch obstruction (*n* = 50)
*r *= 0.65 for class II, two-ventricle heart with arch obstruction (*n* = 15)
*r* = 0.76 for class III, single ventricle heart without arch obstruction (*n* = 10)
*r* = 0.53 for class IV, single ventricle heart with arch obstruction (*n* = 25)”

#### 3.1.2. Main analysis

The analysis of the main objective will be based on the evaluation of the correlation between the age at surgery (in number of days of life) and the main analysis criteria selected, namely the neurodevelopmental outcomes at 24 months assessed using the scores obtained on the Bayley-IV scale. The analysis will be conducted for the entire cohort and separately for each type of complex CHD to define the optimal age for surgery for each complex CHD type. To do this, Pearson's correlation coefficient will be estimated with a 95% confidence interval if the application conditions are met (otherwise, the non-parametric Spearman's rank correlation coefficient will be estimated with a confidence interval of 95%). A ROC curve will be plotted to curve to determine (according to Youden's method) the optimal threshold for the age at surgery that discriminates delayed neurodevelopment / normal neurodevelopment according to the main criterion, i.e. neurodevelopment at 24 months according to the Bayley-IV scale. The first secondary endpoint based on the AIMS scores at 4 months and 12 months will be analyzed using the same previously described strategy. The next secondary endpoint based on the clinical assessment by the child neurologist (neurodevelopment considered normal vs. abnormal) at 4 months, 12 months, and 24 months will be analyzed using a comparison test for quantitative data. A Student's t-test will be used if the application conditions are met; otherwise, the non-parametric Mann-Whitney test will be used.

#### 3.1.3. Secondary analysis

##### 3.1.3.1. White matter injuries

The correlation between the age at surgery (in number of days of life) and the presence of WMIs (yes vs. no) will be assessed by describing and comparing age at surgery according to the presence and absence of WMIs. Non-contrast 3 Tesla MRIs with 3D-T1 weighted and axial T2-weighted sequences and diffusion-weighted images will be performed. Qualitative and quantitative analyses of the MRIs will be done using a lesion score as previously reported in the literature (3, 20, 21); specifically, acquired brain injuries will be characterized as (i) cerebral vascular accidents, (ii) white matter injuries (WMI), (iii) intraventricular hemorrhages (IVH), or (iv) global hypoxic-ischemic injury (HI).

The severity of the injuries will be assessed as follows: 0 = normal, 1 = minimal lesion (minimal WMI and IVH grade I-II), 2 = cerebral vascular accident (any cerebral vascular accident), and 3 = moderate or severe lesion (moderate or severe WMI, IVH III, or HI).

Finally, we will assess the severity of the WMIs using the quartered point system (QPS) (21), the volumetric measurement and the categorical scale described by Miller et al. (20) as normal (no lesion), minimum (≤3 WMI, each <2 mm), moderate (>3 WMI or WMI measuring >2 mm, but <5% of the hemisphere in question), or severe (>5% of the affected hemisphere). The same patient may present several brain injuries, which could also be bilateral. The most severe lesion will be retained for the lesion score.

A comparison test for quantitative data will be used. A Student's t-test will be used if the application conditions are met; the non-parametric Mann-Whitney test will be used otherwise. The correlation between the age at surgery (in number of days of life) and the severity of WMI (four levels of severity, including normal) will be conducted using a comparison test for quantitative data. The analysis of variance will be used if the application conditions are met; otherwise, the non-parametric Kruskal-Wallis test will be used.

These analyses will be conducted on the data collected from the MRIs performed in both the preoperative and postoperative phases.

##### 3.1.3.2. Morbidities

The correlation between age at surgery (in number of days of life) and the occurrence of at least one of the elements of post-op morbidity (yes vs. no) will be assessed by describing and comparing age at surgery according to the presence and absence of a morbidity criterion. A comparison test for quantitative data will be used. The Student's t-test will be used if the application conditions are met; otherwise, the non-parametric Mann-Whitney test will be used. This analysis will be conducted with consideration for each element of morbidity in isolation.

##### 3.1.3.3. Length of hospitalization

The correlation between age at surgery (in number of days of life) and the length of postoperative hospitalization will be assessed by estimating the Pearson's correlation coefficient with a 95% confidence interval if the application conditions are met (otherwise, the non-parametric Spearman's correlation coefficient will be estimated with a 95% confidence interval).

##### 3.1.3.4. Moderating role of demographic and surgical factors

The moderating role of demographic characteristics and pre-, intra-, and postoperative factors on the correlation between age at surgery (in number of days of life) and the various prognostic criteria for neonates with complex CHD indicated above will be analyzed using multivariate analyses. For quantitative data (neuropsychological development assessed using Bayley-IV scale scores, neurodevelopment assessed using AIMS scores, and length of postoperative hospitalization), a multivariate linear regression model will be employed. Each of the criteria previously listed will, in turn, be considered a dependent variable. Age at surgery, the explanatory variable of interest, will be systematically incorporated into the models. The main prognostic factors identified in advance according to an analysis of data from the literature, will also be incorporated into the models. Adjusted beta coefficients will be estimated with a 95% confidence interval. Standardized beta coefficients will also be presented to assess the relative role of each factor in the prognosis.

For qualitative data (neurodevelopment judged as normal vs. abnormal in the neuropediatric clinical assessment, the presence of WMIs, the element of morbidity), a multivariate logistics regression model will be utilized. Each of the criteria previously listed will, in turn, be considered a dependent variable. Age at surgery, the explanatory variable of interest, will be systematically incorporated into the models. The main prognostic factors identified in advance according to an analysis of data from the literature will also be incorporated into the models. Adjusted odds ratios will be estimated with a 95% confidence interval. In order to consider the small sample size, the Firth correction may be applied.

## 4. Discussion

The correlation between surgical timing and long-term neurodevelopmental outcomes in children with complex CHD has not been fully studied. Moreover, currently, there is no consensus regarding the optimal surgical timing for neonates with complex CHD. Our study will allow us to define the optimal time-window within the neonatal period to perform cardiac surgery to reduce postoperative morbidity (i.e., postoperative complications) and optimize the neurodevelopment of infants with complex CHD. We hope our work will facilitate the evaluation of the risk/benefit ratio of the chosen therapeutic strategy.

A better understanding of the mechanisms underlying neurodevelopmental disorders in this population will allow the identification of other modifiable risk factors and the development of new prevention and screening strategies to improve the neurodevelopment and overall health of children and adults.

An added challenge for this field is the recruitment of patients with various types of CHD to define a general optimal age for cardiac surgery and an optimal age for each CHD type and not only for TGA and HLHS, which have been studied in more depth.

Moreover, the patients in our study will receive a neurodevelopmental follow-up at 4 months, 12 months, and 24 months. They will benefit from early screening for neurodevelopmental disorders with an orientation toward adapted rehabilitation structures, thereby supporting improvements in their overall neurodevelopmental outcome. The aim is to extend this treatment process to all patients with complex CHD who undergo surgery in their first weeks of life. This project, initially internal in hospitals in Marseille, could be extended with the collaboration of pediatric cardiology and neurology teams across France.

## 5. Limitation

As Complex CHD are characterized by their heterogeneity, the monocentric nature of our protocol study may be considered a limitation, as well as the relatively low number of participants to be recruited (*n* = 100). This preliminary work may allow us to define an optimal surgery timing only for those CHDs that recruit frequently (e.g., TGA, aortic arch hypoplasia). The extension of this project to other pediatric cardiology and neurology teams in France will enable more reliable results to be obtained.

## 6. Conclusion

We will conduct a preliminary observational study to determine the most appropriate surgical timing for each type of complex CHD. This project aims to determine whether an earlier surgery would be a beneficial neuroprotective strategy to reduce brain injuries and postoperative morbidities and improve neurodevelopmental outcomes. This research should be useful in developing guidelines for optimal surgical timing for complex CHDs in the near future. Based on the balance between benefit and risk, these guidelines will make it easier to determine the optimal surgical timing for a newborn or infant with a complex CHD. Secondly we will conduct an interventional study to reduc the age at surgery.
